# Evidence for Previously Unidentified Sexual Transmission of Protozoan Parasites

**DOI:** 10.3201/eid2403.171838

**Published:** 2018-03

**Authors:** Clara Crespillo-Andujar, Marta Díaz-Menéndez, Marta Mora-Rillo

**Affiliations:** National Referral Unit for Tropical and Travel Medicine, Hospital Universitario la Paz-Carlos III, Madrid, Spain (C. Crespillo-Andujar, M. Díaz-Menéndez);; Hospital Universitario la Paz-Carlos III, Madrid (M. Mora-Rillo)

**Keywords:** Viruses, parasites, protozoa, semen, sexual transmission, seminal fluid, Entamoeba hystolitica, Schistosoma haematobium, Trichomonas vaginalis, Trypanosoma cruzi, Toxoplasma gondii, urethra, testis, epididymis, urogenital, parasite, protozoan, seminal vesicles, prostate, infertility

## Abstract

Knowing the mode of transmission of a disease can affect its control and prevention. Here, we identify 5 protozoan parasites with demonstrated presence in seminal fluid, only 1 of which has been identified as a sexually transmitted disease among humans.

A recent publication by Salam and Horby (1 (*1*) identified at least 27 viruses present in human semen, some potentially transmissible through sexual contact. Trichomonas vaginalis is a protozoan parasite recognized as sexually transmissible among humans (*2*). Similar to that which occurs with viruses, parasites could reach seminal fluid by passing from the bloodstream to the male genital tract or by directly infecting reproductive organs. In this context, more parasitic infections might also be transmitted sexually. Considering that parasitic diseases represent one of the most common infections worldwide, mainly in developing countries, sexual transmission of parasitic diseases could represent a major global problem in terms of public health.

To investigate whether parasites could enlarge the broad list of potential sexually transmitted infections (STIs), we conducted an online search on November 3, 2017, by using PubMed (https://www.ncbi.nlm.nih.gov/pubmed/), EMBASE (https://www.elsevier.com/solutions/embase-biomedical-research), and the Cochrane Library (http://www.cochranelibrary.com/) with no language restrictions. We used the terms “parasites OR parasitic disease” and “semen OR seminal plasma.” We also made a manual search of the references of selected reports. Two reviewers independently screened the 512 returned results of titles, abstracts, and full text in selected articles.

Our search resulted in 5 parasite species with demonstrated presence in seminal fluid of humans: *Entamoeba* *hystolytica* (*3*), *Schistosoma* *haematobium* (*4*), *Trichomonas* *vaginalis* (*2*), *Trypanosoma* *cruzi* (*5*), and *Toxoplasma* *gondii*; the latter has been documented as sexually transmitted among animals, but not humans (*6*) (Table). *E*. *hystolytica* is a worldwide anaerobic protozoan; its prevalence increases disproportionately in areas of poor sanitation in low-income countries. *E*. *hystolitica* has been identified in the testicles, epididymis, and seminal fluid (*3*,*7*), can reportedly cause infertility as a result of reproductive organ damage (*8*), and is transmitted by sexual contact (both oral-anal and oral-genital sexual practices) (*7*).

**Table:** Results of literature search for reports of parasites found in semen samples

Parasite (reference)	Method for parasite identification	Type of study	Evidence of sexual transmission	Evidence of causing infertility
*Entamoeba hystolytica* (*2*)	Microscopy	Case report	In humans	In humans
*Schistosoma haematobium* (*3*)	Microscopy	Cohort study	No evidence	In humans
*Trichomonas vaginalis* (*4*)	PCR	Cohort study	In humans	In humans
*Trypanosoma cruzi* (*5*)	PCR	Cohort study	Experimental mouse model	In animals
*Toxoplasma gondii* (*6*)	Microscopy	Cohort study	Experimental sheep model	In humans

Urogenital schistosomiasis caused by *S*. *haematobium* infection affects male and female children and adults mainly in Africa, the Middle East, and Corsica, France. After the larval *S*. *haematobium* cercarias penetrate intact skin from contaminated fresh water, they migrate and mature into adult worms, predominantly in the venous plexus of the bladder. These worms can then travel to the seminal vesicles and prostate, causing local pathology (*9*). *S*. *haematobium* eggs have been found in up to 43% of 44 semen samples and in 33.3% of cervix biopsies obtained from 36 women from endemic area populations (*4*,*10*); nevertheless, sexual transmission has not been reported.

*T.* *vaginalis* protozoa are the most common nonviral STI in the world, and incidence is increasing (*11*). The genital tract of humans is the natural habitat for this parasite, which can cause urogenital tract infection. *T*. *vaginalis* has been identified in seminal fluid and has been related to decreased sperm quality (*2*,*8*).

Chagas disease is caused by *T.* *cruzi* protozoa and affects nearly 6 million persons in Latin America countries. Parasitic involvement of the male genital tract, alteration in semen characteristics (*7*), and the presence of the parasite’s DNA in semen have recently been identified in chronically infected patients (*5*)*.* Furthermore, an experimental mouse model has demonstrated infection through intravaginal infusion of semen from infected humans, posing the possibility of sexual transmission among humans (*5*).

Toxoplasmosis is a protozoan disease caused by *T.* *gondii* infection, with a worldwide prevalence from 20% to 80%. *T*. *gondii* has been found in the semen of infected men (*6*), and infection has been related to a decrease in semen quality (*8*). An experimental sheep model demonstrated infection after vaginal infusion of vegetative cysts of *T*. *gondii* (*7*).

Despite evidence of the presence of parasites in semen, the specific mechanism by which the parasite reaches the semen has not been clearly elucidated. The male genital tract can be invaded through a connection from the urethra to the testis or epididymis (*T.* *vaginalis*, *S*. *haematobium*), invasion from an adjacent structure (*E.* *hystolytica*), or as a result of a disseminated infection (*T.* *cruzi*, *T.* *gondii*) (*7*).

Other parasites, such as *Plasmodium* spp. and *Trypanosoma* *brucei*, can affect human spermatogenesis and impair fertility (*7*,*8*). *Leishmania* spp. have been detected in the testes in autopsies of patients who died of visceral leishmaniasis, and infection with these species has induced infertility (*8*). Although the presence of the *Leishmania*, *T*. *brucei*, and *Plasmodium* spp. in the male genital tract have been demonstrated, they have not been detected in human semen (*7*).

According to our review, it might be crucial to consider the sexual route of infection among parasitic diseases with seminal presence in addition to viral diseases previously identified (*1*). Infertility might be a consequence of parasitic infection regardless of its presence in seminal fluid (*8*).

These parasitic infections primarily affect vulnerable populations in developing countries. Many of these diseases are classified as neglected tropical diseases because of the scarcity of resources for their study; thus, potential sexual transmission of them is underresearched. The lack of available scientific information about the role of parasites in genital fluids leaves room for confusion about the relative importance of sexual activity as a route of transmission. Further studies are needed to implement better public health strategies.
